# Quality Improvement Study: Referral to Cardiac Rehabilitation After Percutaneous Coronary Intervention

**DOI:** 10.7759/cureus.100584

**Published:** 2026-01-01

**Authors:** Natalia Fongrat, Geronimo Guzman, Courtney Deihr, Saniya Mohsin, Anna Tomdio, Sebhat Erqou, Luis A Guzman

**Affiliations:** 1 Internal Medicine, Mary Washington Hospital, Fredericksburg, USA; 2 Medicine, Saba University School of Medicine, Saba, NLD; 3 Medicine, Mary Washington Hospital, Fredericksburg, USA; 4 Cardiology, Mary Washington Hospital, Fredericksburg, USA

**Keywords:** cardiac rehabilitation, coronary artery intervention, pci, quality improvement projects, quality metrics

## Abstract

Background

Cardiac rehabilitation (CR) is a key post-percutaneous coronary intervention (PCI) quality metric and is known to improve cardiovascular outcomes. Although referral rates after PCI have been described, far less is known about what occurs after a referral order is placed, specifically whether patients are successfully contacted, whether they enroll, and the barriers they encounter. This quality improvement (QI) project aimed to determine referral and enrollment rates following PCI, compare referral timing at discharge versus the first follow-up visit, and identify patient-reported and system-level barriers contributing to low CR participation.

Methods

We performed a retrospective chart review of 200 consecutive patients who were at least three months post-percutaneous coronary intervention. All patients not enrolled in the CR program at our center were prospectively contacted at home to confirm if they were referred to, and whether they were doing CR. If patients were not enrolled, reasons were explored.

Results

A total of 194 patients were discharged. The mean age was 68±11 years, and 34% (n=65) were female. The overall referral rate was 55% (n=107), including 21.6% (n=42) at hospital discharge and 33.5% (n=65) at the first outpatient clinic visit. After at least three months post-PCI, only 35 patients (18% of the entire group and 33% of those referred) were enrolled in CR. Of those with a referral order placed at discharge, only 10 (24%) were doing CR. The most common reason for non-enrollment in cardiac rehabilitation was lack of awareness of the need to enroll, reported by 58% (n=92) of respondents. In a multivariate analysis, PCI in an urgent setting and prior PCI were the only independent predictors of non-referral. Not private insurance and diabetes were independent predictors of lack of CR participation.

Conclusions

Referral to, and especially enrollment in, the CR programs post-PCI remained markedly low. Enrollment tended to be lower when referrals were placed at discharge, highlighting limitations of current quality metrics that focus solely on referral placement. Improving patient and physician education and strengthening system-level processes may help support higher enrollment and more effective use of CR.

## Introduction

Percutaneous coronary intervention (PCI) has significantly improved survival and symptom relief in patients with coronary artery disease, but optimal long-term management requires consistent lifestyle modification, medication adherence, and participation in cardiac rehabilitation (CR) [1-5]. CR is a comprehensive, multidisciplinary intervention that integrates exercise training, lifestyle counseling, psychosocial support, and education to reduce recurrent cardiovascular events and improve functional capacity [2,6]. Despite these well-established benefits, participation in CR remains low nationwide, particularly among patients undergoing PCI [1-4].

Previous studies have largely focused on referral rates, while the steps that occur after a referral is placed have been far less examined [7,8]. Important aspects such as whether the referral reaches the rehabilitation center, whether patients are contacted, and whether they enroll or decline CR are rarely evaluated in detail. These post-referral processes represent critical gaps that likely contribute to persistently low participation rates and remain insufficiently characterized in routine clinical practice.

At our institution, data from the National Cardiovascular Data Registry (NCDR) revealed that referral rates after PCI were substantially lower than national benchmarks, prompting the development of this quality improvement project. An additional observation was that many interventional cardiology providers preferred to place CR referrals during the first follow-up clinic visit rather than at hospital discharge. This difference raised the possibility that the timing of the referral may influence patient readiness, comprehension of the program, or the quality of patient-provider engagement, all of which could affect enrollment.

The purpose of this project was to examine the full continuum of CR referral and enrollment after PCI, with particular attention to the challenges that arise after a referral order is placed. The study also sought to explore differences between referrals made at discharge and those made during follow-up, and to identify patient-reported and system-level barriers that interfere with enrollment. By evaluating these factors, we aimed to better understand how current processes affect participation and how they align with existing national quality metrics, which primarily emphasize placing the referral order rather than ensuring successful enrollment. This project provides a clearer view of real-world barriers within an entire post-PCI population and highlights several opportunities to improve CR utilization.

## Materials and methods

This quality improvement project was conducted at a single community hospital in the United States and included both retrospective electronic chart review and prospective patient interviews. Patients who underwent PCI between March 2024 and June 2024 at Mary Washington Hospital in Fredericksburg, Virginia, were identified, and consecutive adults with at least three months of follow-up at the time of data collection were included. This follow-up period allowed adequate time for referral processing, patient contact, and potential enrollment into a CR program. Patients who died before discharge, were transferred to another facility for advanced care, or lacked follow-up information were considered ineligible for assessment of CR referral or enrollment.

Patient selection began by identifying all individuals who underwent PCI within the study period through procedure codes and interventional cardiology case logs. The electronic health record (EHR) system (EPIC) was used to extract demographic information, clinical characteristics, procedural indications, in-hospital data, and post-discharge documentation relevant to CR referral and enrollment. Two reviewers independently examined each chart to determine whether a referral to CR was placed, the timing of the referral, and the information available on CR program contact attempts. Referral was defined as the placement of a formal CR referral order in the EHR directing the rehabilitation center to contact the patient. Contact was defined as a documented attempt by the CR program to reach the patient via telephone or electronic message. Enrollment was defined as the patient attending at least one CR session at the designated center. Participation referred to ongoing involvement beyond the first session but was not formally assessed in this project.

To enhance reproducibility, the process of extracting information from the EHR followed a consistent sequence for each patient. Reviewers first confirmed whether a referral order existed, then examined discharge summaries, clinic notes, and rehabilitation center documentation to determine whether the order was transmitted, whether the patient was contacted, and whether communication attempts were successful. Details such as the number of call attempts, full or unopened voicemail boxes, and insurance authorization delays were recorded when available. Discrepancies between reviewers were resolved through consensus and, when necessary, by involving a third investigator.

Prospective telephone interviews were conducted for patients who had not enrolled in CR, in order to verify whether they had been referred and to better understand reasons for non-participation. Three authors who were familiar with CR processes performed the interviews after receiving standardized instruction on how to deliver questions consistently. A uniform script was used to minimize interviewer variability and included prompts to assess awareness of the referral, understanding of CR, logistical barriers, insurance concerns, physical limitations, and personal preferences.

A brief provider survey was also conducted to assess perceived barriers to placing CR referral orders. An electronic questionnaire was distributed by email to all eight interventional cardiology providers over a two-week period. The survey included items addressing workflow challenges, timing preferences for referral placement, perceived patient readiness, and other factors influencing referral decisions. Responses were collected anonymously, and the response rate was 100%. Survey results were summarized descriptively and used to complement the findings from the chart review.

Missing data were handled through complete-case analysis. If essential variables such as referral status, enrollment outcome, or patient contact information were unavailable despite a thorough EHR review, the patient was excluded from specific analyses but retained in overall descriptive reporting when possible. Because this was a quality improvement project designed to characterize real-world practices, no imputation procedures were applied. Missingness for key variables was minimal and did not affect the overall analytic strategy.

Statistical analysis was performed using Stata version 15 (StataCorp, College Station, TX). Continuous variables were summarized as means with standard deviations, and categorical variables as counts with percentages. Comparisons between groups were made using Student's t-tests for continuous variables and Pearson's chi-square tests for categorical variables. Multivariable logistic regression was conducted to identify independent predictors of referral and enrollment. Age and sex were forced into the models based on clinical relevance. Remaining variables were considered for inclusion using a forward stepwise approach, with entry and removal thresholds defined a priori, consistent with exploratory quality improvement (QI) methodology. Odds ratios with 95% confidence intervals were calculated, and statistical significance was defined as a two-sided p-value less than 0.05. Model assumptions were examined, and collinearity among covariates was assessed before finalizing the regression models.

This project was reviewed by the hospital's Quality Improvement Committee and determined to be a quality improvement initiative, exempt from IRB oversight under institutional policy.

## Results

**Figure 1:**
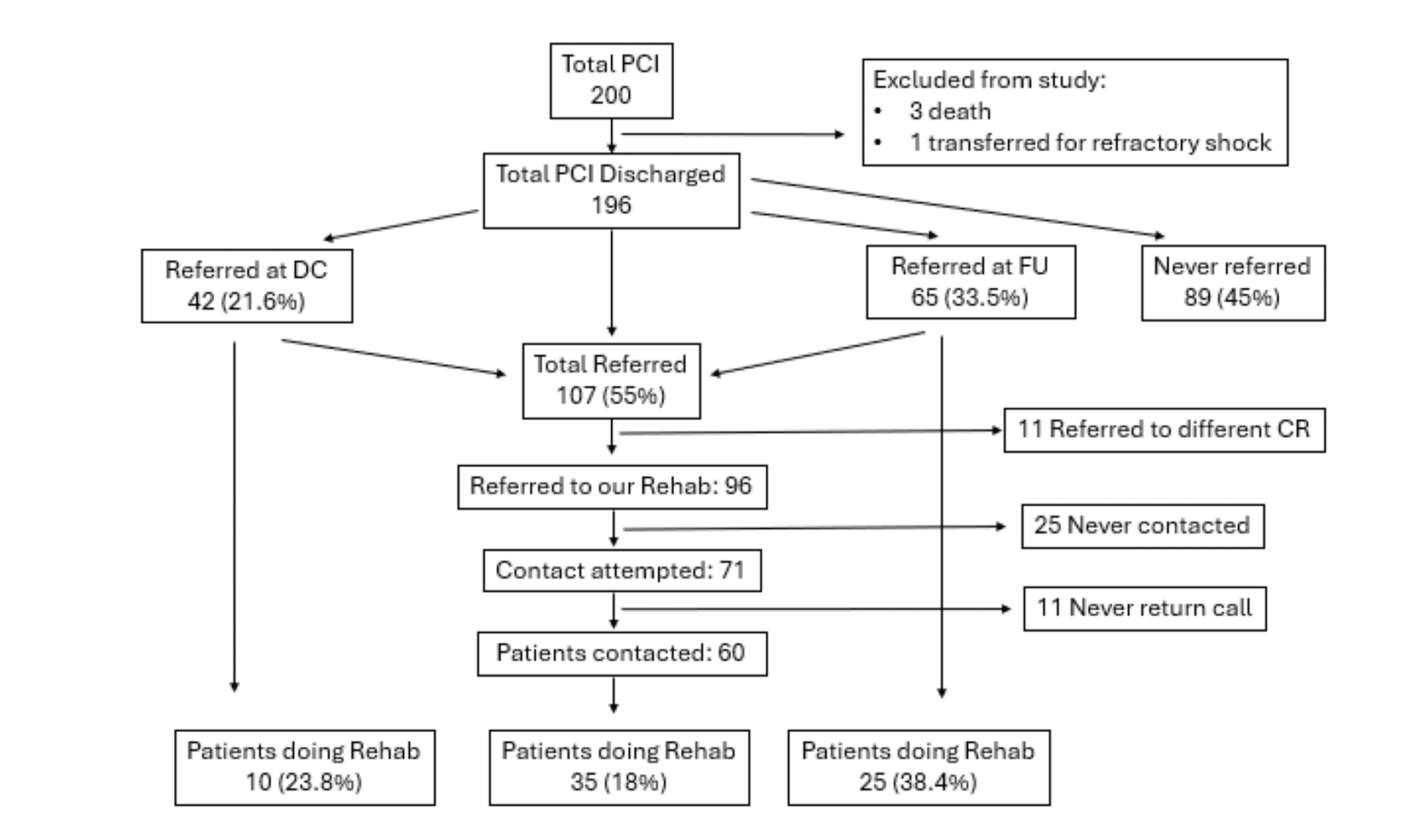
Flowchart of the cardiac rehabilitation referral and enrollment process following percutaneous coronary intervention CR - cardiac rehabilitation; PCI - percutaneous coronary intervention; DC - discharge; FU - follow-up

Of the 194 eligible patients, 107 (55%) received a referral to cardiac rehabilitation. Only 42 patients (21.6%) were referred at the time of hospital discharge, while 65 (33.5%) were referred during their first follow-up clinic visit (Figure 1). This flowchart illustrates the referral and enrollment pathway for 200 patients who underwent PCI. Of these, 196 patients were discharged alive, while four were excluded due to death or transfer for refractory shock. A total of 107 patients (55%) were referred to CR - 42 (21.6%) at hospital discharge, and 65 (33.5%) during follow-up. Eleven patients were referred to a different CR center, and 25 (23%) were never contacted despite referral. Among the 71 patients successfully contacted by the rehabilitation center, 35 (50%) enrolled in CR, representing 18% of the total PCI cohort. Enrollment was higher among those referred during follow-up (38.4%) compared with those referred at discharge (23.8%), highlighting gaps in post-discharge communication and patient engagement. The provider survey revealed that most interventional cardiologists cited the complexity of placing the referral order at discharge as the primary barrier, and several indicated a preference for discussing CR during the first clinic visit, when patients were more receptive to education.

**Figure 2:**
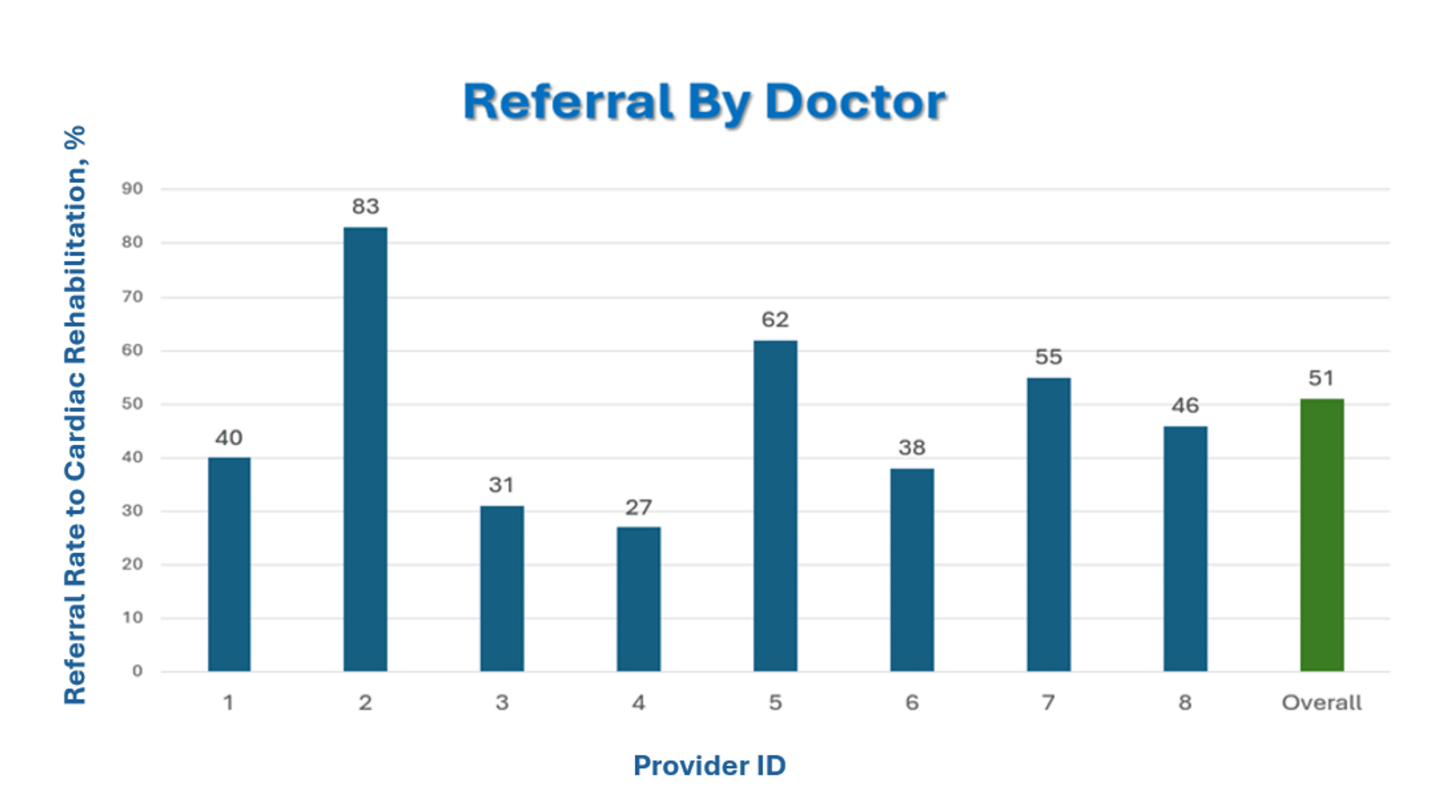
Referral rate to CR by provider following PCI CR - cardiac rehabilitation; PCI - percutaneous coronary intervention

Referral rates also varied across operators. Figure 2 illustrates the percentage of patients referred to CR after PCI, stratified by provider (1-8). Referral rates varied among providers, ranging from 27% to 83%, with an overall institutional average of 51% (n=107). Despite some variability between providers, the differences were not statistically significant, suggesting that system-level factors, rather than provider-specific practices, contribute most to the low overall referral rate. As shown in Figure 2, the spread in individual provider referral percentages was modest and did not reach statistical significance, supporting the interpretation that system-level processes, rather than individual practice patterns, were the dominant factors contributing to low referral rates. Multivariable analysis confirmed that urgent PCI and prior PCI were associated with a lower likelihood of referral, whereas age and sex showed no independent association.

Enrollment into CR was considerably lower than the referral rate. Of the 107 patients referred, nearly one-third had no documented contact from the CR center, either because calls were not made, contact information was outdated, or repeated attempts were unsuccessful. Among the 71 patients who were successfully reached, 35 ultimately enrolled, representing 50% of those contacted and only 18% of the entire PCI cohort. Enrollment was higher for patients referred during the first follow-up visit compared to those referred at discharge, although this difference did not reach statistical significance. Patients with private insurance were more likely to enroll than those with Medicare or Medicaid, and those without a history of prior PCI had higher enrollment than patients with previous revascularization. Diabetes and peripheral artery disease were associated with lower enrollment in unadjusted analyses, with diabetes remaining an independent predictor in multivariable modeling. Age and sex again showed no significant association with enrollment.

Reasons for non-participation were obtained from 139 of the 159 patients who did not enroll. Lack of awareness about CR was the most commonly reported barrier overall, followed by physical limitations, personal preference or declining participation, logistical challenges such as transportation or scheduling conflicts, and insurance-related issues. When stratified by timing of referral, the distribution of barriers differed substantially. Patients referred at discharge most frequently reported being unaware of the referral or not understanding the purpose of CR, whereas those referred at the follow-up visit more often cited functional limitations or logistical concerns. These findings suggest that the context and timing of the referral influence patient comprehension and subsequent engagement with CR.

Table 1 summarizes the patient baseline characteristics of the study population stratified by CR referral status. The mean age of the entire group was 68±11 years, and 34% (n=65) were female. Elective PCI accounted for 57% (n=110) of procedures, while 13% (n=26) of patients underwent PCI for STEMI. Other than hypertension and prior revascularization (PCI or coronary artery bypass graft, CABG), there were no significant differences in baseline clinical characteristics among those referred and those not referred to CR.

**Table 1:** Baseline demographic, clinical, and procedural characteristics of 194 patients undergoing PCI, stratified by CR referral status Continuous variables are expressed as mean±SD and categorical variables as number and percentage. Group comparisons between enrolled and non-enrolled patients were performed using the Student's t-test for continuous variables and Pearson's chi-square test for categorical variables. CR - cardiac rehabilitation; PCI - percutaneous coronary intervention; CABG - coronary artery bypass graft; PAD - peripheral artery disease; HTN - hypertension; HLD - hyperlipidemia; DM - diabetes mellitus; COPD - chronic obstructive pulmonary disease; CVA - cerebrovascular accident; LVEF - left ventricular ejection fraction; HF - heart failure; STEMI - ST-segment elevation myocardial infarction; NSTEMI - non-ST-segment elevation myocardial infarction; MI - myocardial infarction

Variable	Overall (n=194), n (%)	Referred (n=107), n (%)	Not referred (n=87), n (%)	p-value
Age, mean±SD	68±11	68±10	67±11	0.487
Gender	194 (100%)	107 (55%)	87 (45%)	0.964
Female	65 (34%)	36 (34%)	29 (33%)	
Male	129 (66%)	71 (66%)	58 (67%)	
PCI status	194 (100%)	107 (55%)	87 (45%)	0.042
Elective	110 (57%)	67 (63%)	43 (49%)	
Emergency	26 (13%)	16 (15%)	10 (12%)	
Urgent	58 (30%)	24 (22%)	34 (39%)	
Inpatient/outpatient	194 (100%)	107 (55%)	87 (45%)	0.065
In	84 (43%)	40 (37%)	44 (51%)	
Out	110 (57%)	67 (63%)	43 (49%)	
Health insurance	194 (100%)	107 (55%)	87 (45%)	0.154
Medicaid	20 (11%)	7 (7%)	13 (15%)	
Medicare	115 (59%)	67 (63%)	48 (55%)	
Private	59 (30%)	33 (30%)	26 (30%)	
HTN	194 (100%)	107 (55%0	87 (45%)	0.078
No	22 (11%)	16 (15%)	6 (7%)	
Yes	172 (89%)	91 (85%)	81 (93%)	
HLD	194 (100%)	107 (55%)	87 (45%)	0.337
No	18 (9%)	8 (7%)	10 (12%)	
Yes	176 (91%)	99 (93%)	77 (88%)	
DM	194 (100%)	107 (55%)	87 (45%)	0.227
No	118 (61%)	61 (57%)	57 (66%)	
Yes	76 (39%)	46 (43%)	30(34%)	
Tobacco use	190 (98%)	105 (55%)	85 (45%)	0.194
Current	41 (22%)	19 (18%)	22 (26%)	
Other	149 (78%)	86 (82%)	63 (74%)	
COPD	194 (100%)	107 (55%)	87 (45%)	0.309
No	164 (85%)	93 (87%)	71 (81%)	
Yes	30 (15%)	14 (13%)	16 (19%)	
PAD	194 (100%)	107 (55%)	87 (45%)	0.156
No	162 (84%)	93 (87%)	69 (79%)	
Yes	32 (16%)	14 (13%)	18 (31%)	
Prior MI	194 (100%)	107 (55%)	87 (45%)	0.103
No	153 (79%)	89 (83%)	64 (74%)	
Yes	41 (21%)	18 (17%)	23 (26%)	
Prior PCI	194 (100%)	107 (55%)	87 (45%)	0.004
No	111 (57%)	71 (66%)	40 (46%)	
Yes	83 (43%)	36 (34%)	47 (54%)	
Prior CABG	194 (100%)	107 (55%)	87 (45%)	0.229
No	175 (90%)	99 (93%)	76 (87%)	
Yes	19 (10%)	8 (7%)	11 (13%)	
CVA	194 (100%)	107 (55%)	87 (45%)	0.801
No	150 (77%)	82 (77%)	68 (78%)	
Yes	44 (23%)	25 (23%)	19 (22%)	
LVEF, mean±SD	N/A	53±9	49±13	0.127
HF	194 (100%)	107 (55%)	87 (45%)	0.028
No	138 (71%)	83 (78%)	55 (63%)	
Yes	56 (29%)	24 (22%)	32 (37%)	

Table 2 summarizes demographic characteristics, cardiovascular risk factors, comorbidities, procedural urgency, and insurance type, stratified by post-procedure CR enrollment status. The table outlines the characteristics of patients who enrolled in cardiac rehabilitation (CR) compared with those who did not. Individuals with private insurance were nearly four times more likely to participate in CR than those covered by Medicaid, and enrollment was significantly higher among patients without a history of prior PCI. There was also a notable trend toward lower enrollment among older patients, those referred at discharge, individuals with diabetes, and those with a history of peripheral artery disease (PAD).

**Table 2:** Baseline demographic, clinical, and procedural characteristics of 194 patients undergoing PCI, stratified by post-procedure CR enrollment status Continuous variables are expressed as mean±SD and categorical variables as number and percentage. Group comparisons between enrolled and non-enrolled patients were performed using the Student's t-test for continuous variables and Pearson's chi-square test for categorical variables; MI - myocardial infarction CR - cardiac rehabilitation; PCI - percutaneous coronary intervention; CABG - coronary artery bypass graft; PAD - peripheral artery disease; HTN - hypertension; HLD - hyperlipidemia; DM - diabetes mellitus; COPD - chronic obstructive pulmonary disease; CVA - cerebrovascular accident; LVEF - left ventricular ejection fraction; HF - heart failure; STEMI - ST-segment elevation myocardial infarction; NSTEMI - non-ST-segment elevation myocardial infarction

Variables	Overall (n=194), n (%)	Enrolled (n=35), n (%)	Not enrolled (n=159), n (%)	p-value
Age, mean±SD	68±11	65±8	68±11	0.11
Gender	194 (100%)	35 (18%)	159 (82%)	0.914
Female	65 (34%)	12 (34%)	53 (33%)	
Male	129 (66%)	23 (66%)	106 (67%)	
PCI status	194 (100%)	35 (18%)	159 (82%)	0.028
Elective	110 (57%)	20 (57%)	90 (57%)	
Emergency	26 (13%)	9 (26%)	17 (11%)	
Urgent	58 (30%)	6 (17%)	52 (32%)	
Inpatient/outpatient	194 (100%)	35 (18%)	159 (82%)	0.954
In	84 (43%)	15 (43%)	69 (43%)	
Out	110 (57%)	20 (57%)	90 (57%)	
Health insurance	194 (100%)	35 (18%)	159 (82%)	0.035
Medicaid	20 (10%)	3 (9%)	17 (11%)	
Medicare	115 (59%)	15 (43%)	100 (63%)	
Private	59 (3%1)	17 (48%)	42 (26%)	
HTN	194 (100%)	35 (18%)	159 (82%)	0.232
No	22 (11%)	6 (17%)	16 (10%)	
Yes	172 (89%)	29 (83%)	143 (90%)	
HLD	194 (100%)	35 (18%)	159 (82%)	0.873
No	18 (9%)	3 (9%)	15 (9%)	
Yes	176 (91%)	32 (91%)	144 (91%)	
DM	194 (100%)	35 (18%)	159 (82%)	0.3
No	118 (61%)	24 (69%)	94 (59%)	
Yes	76 (39%)	11 (31%)	65 (41%)	
Tobacco use	190 (98%)	34 (18%)	156 (82%)	0.194
Current	41 (22%)	5 (15%)	36 (23%)	
Other	149 (77%)	29 (85%)	120 (77%)	
COPD	194 (100%)	35 (18%)	159 (82%)	0.213
No	164 (85%)	32 (91)	132 (83)	
Yes	30 (15%)	3 (9)	27 (17)	
PAD	194 (100%)	35 (18)	159 (82)	0.058
No	162 (84%)	33 (94%)	129 (81%)	
Yes	32 (16%)	2 (6%)	30 (19%)	
Prior MI	194 (100%)	35 (18%)	159 (82%)	0.12
No	153 (73%)	31 (89%)	122 (77%)	
Yes	41 (21%)	4 (11%)	37 (23%)	
Prior PCI	194 (100%)	35 (18%)	159 (82%)	0.024
No	111 (57%)	26 (74%)	85 (53%)	
Yes	83 (43%)	9 (26%)	74 (47%)	
Prior CABG	194 (100%)	35 (18%)	159 (82%)	0.127
No	175 (90%)	34 (97%)	141 (89%)	
Yes	19 (10%)	1 (3%)	18 (11%)	
CVA	194 (100%)	35 (18%)	159 (82%)	0.19
No	150 (77%)	30 (86%)	120 (75%)	
Yes	44 (23%)	5 (14%)	39 (25%)	
LVEF, mean±SD	122±63	21±17	101±83	0.26
HF	194 (100%)	35 (18%)	159 (82%)	0.201
No	138 (71%)	28 (80%)	110 (69%)	
Yes	56 (29%)	7 (20%)	49 (31%)	
HF type	56 (29%)	7 (13%)	49 (88%)	0.266
Diastolic	27 (48%)	2 (29%)	25 (51%)	
Systolic	29 (52%)	5 (71%)	24 (49%)	
Timing to order	107 (55%)	35 (33%)	72 (67%)	0.11
At discharge	42 (39%)	10 (29%)	32 (44%)	
1^st^ appointment	65 (61%)	25 (71%)	40 (56%)	

**Figure 3:**
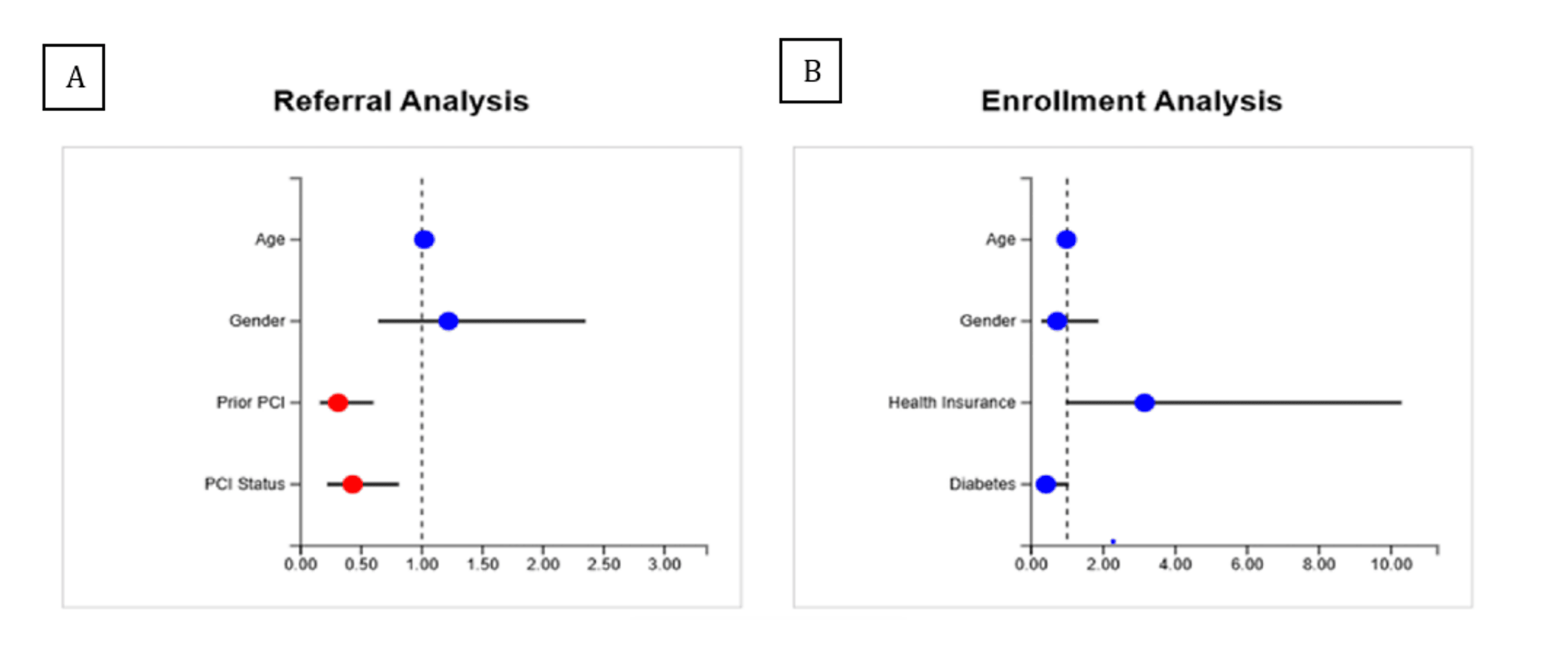
Stepwise multivariable logistic regression of factors associated with CR referral and enrollment after PCI Panel A (referral analysis): forest plot displaying odds ratios (ORs) with 95% confidence intervals (CIs) for predictors of being referred to CR. Panel B (enrollment analysis): forest plot displaying ORs (95% CIs) for predictors of enrolling in CR among referred patients. Stepwise regression model: age and gender were forced into both models. A forward stepwise approach was used with entry p<0.10 and removal p≥0.20. ORs and 95% CIs were estimated using logistic regression. The dashed vertical line at OR=1.0 indicates no effect. Red dots denote statistically significant variables (p<0.05); blue dots denote variables not reaching statistical significance (p≥0.05).

The regression analyses summarizing independent predictors of referral and enrollment are presented in Figure 3. Points to the left of 1.0 indicate decreased odds; to the right of 1.0 indicate increased odds of the outcome (referral or enrollment). In the referral model, factors such as prior PCI and PCI status showed significant associations with referral likelihood. In the enrollment model, health insurance status and diabetes were the strongest effects, consistent with lower enrollment among patients without private insurance and those with diabetes.

**Figure 4:**
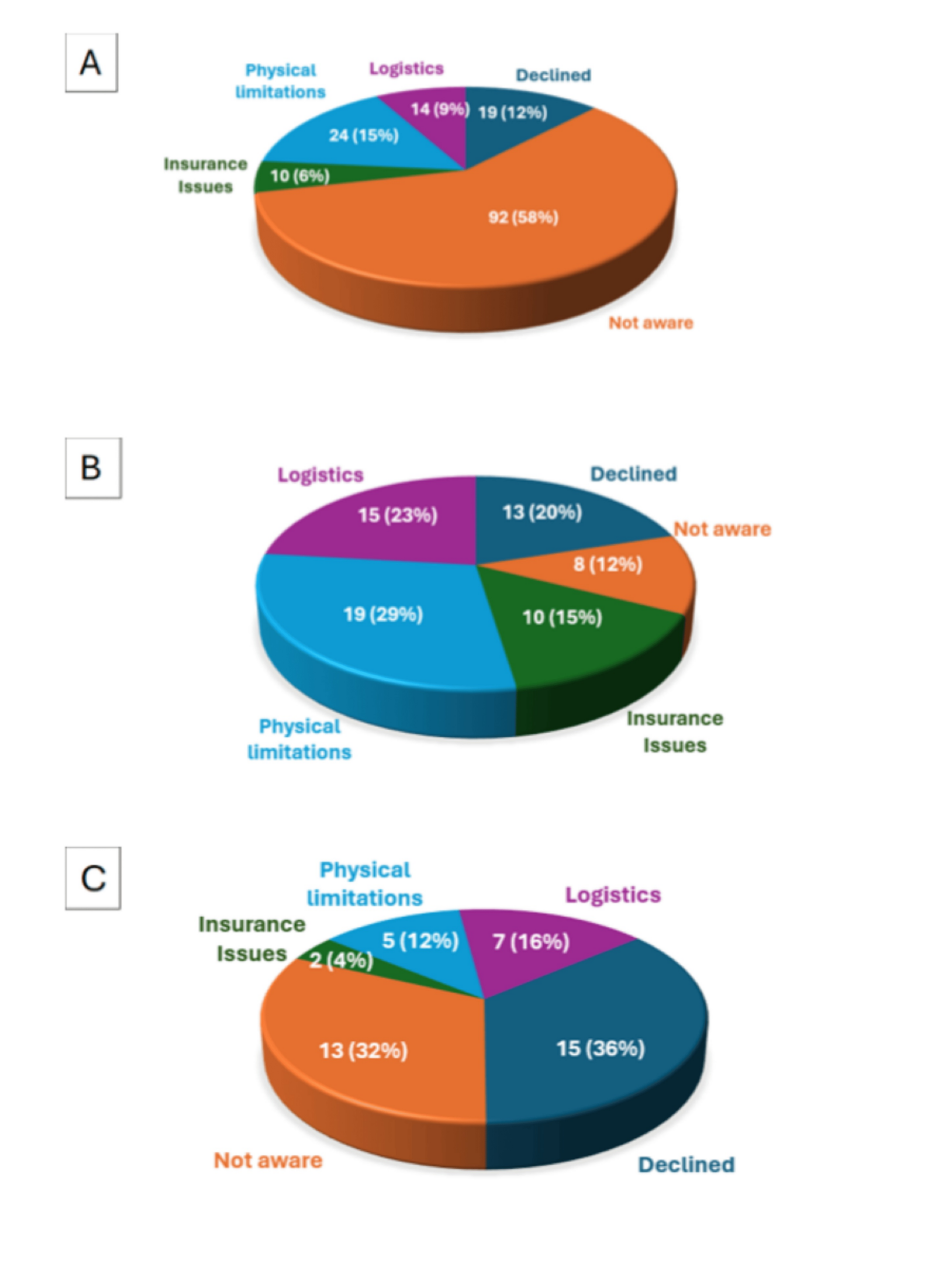
Reasons for non-participation in CR after PCI Panel A: overall distribution of reasons for non-enrollment in CR among patients who were eligible but did not participate. Panel B: Reasons for non-enrollment among patients referred to CR at the first follow-up clinic visit. Panel C: Reasons for non-enrollment among patients referred to CR at hospital discharge. CR - cardiac rehabilitation; PCI - percutaneous coronary intervention

Patient-reported barriers are displayed in Figure 4. An overall distribution of reasons for non-enrollment in CR among patients who were eligible but did not participate (Figure 4A) shows that the most common barrier was lack of awareness about the program 58% (n=92), followed by physical limitations 15% (n=24), declined participation 12% (n=19), logistical challenges 9% (n=14), and insurance issues 6% (n=10). Reasons for non-enrollment among patients referred to CR at the first follow-up clinic visit (Figure 4B) show that the most frequent barriers were physical limitations 29% (n=19) and logistics 23% (n=15), while lack of awareness was reported in only 12% (n=8) of cases, suggesting improved patient understanding when CR was discussed during follow-up. Reasons for non-enrollment among patients referred to CR at hospital discharge (Figure 4C) were lack of awareness 32% (n=13), and declined participation 36% (n=15), with fewer citing logistics 16% (n=7), physical limitations, 12% (n=5), or insurance issues, 4% (n=2). Overall, lack of awareness and logistical barriers were the predominant reasons for low CR participation, emphasizing the importance of structured patient education and coordinated follow-up to improve post-PCI enrollment.

## Discussion

This quality improvement project highlights significant challenges in the referral and enrollment processes for cardiac rehabilitation (CR) following percutaneous coronary intervention (PCI). Although slightly more than half of the cohort received a CR referral (n=107), referral placement at the time of discharge remained particularly low. Enrollment was exceedingly limited, with fewer than 20% of all eligible patients (n=35) ultimately participating, and only one-third of those referred completing the enrollment process. These results illustrate several breakdowns across the referral-to-enrollment pathway and emphasize the multilevel nature of the barriers identified. One of the most consistent findings was the widespread lack of patient awareness regarding the purpose and availability of CR. Additionally, the analysis did not identify strong or consistent associations between baseline clinical characteristics and either referral or enrollment, suggesting that system-level challenges may be the predominant drivers of low participation.

Placing the referral order at discharge

Placing a referral order at discharge has been reported as a key factor in improving CR enrollment. A Canadian study demonstrated that automatic referral at discharge resulted in 60% enrollment compared with 29% when referral relied on physician discretion [9,10]. The low rate of discharge referrals in this project was a central motivator for initiating this QI effort and raises concern given its importance as a hospital quality metric and its inclusion in the Centers for Medicare and Medicaid Services (CMS) performance measures since 2014 [11]. Providers most often cited the complexity of placing the referral order as the primary reason for low referral rates, a finding that aligns with prior work highlighting the challenges of EHR navigation and administrative burden [10,12-16]. Although a system of automatic referral placement could address this barrier, as demonstrated in several institutions, it is notable that enrollment remained limited even among those referred at discharge. Many providers felt that the first follow-up visit offered a more appropriate opportunity to engage patients, and while this approach does not meet the discharge referral metric, it may facilitate better patient understanding of CR. Prior studies have shown that education regarding CR can significantly improve both referral and enrollment rates [10,17,18]. Our findings support this observation, as patients referred at discharge frequently reported unawareness of the program or lack of clarity about its purpose, with nearly two-thirds expressing these concerns.

Logistics involved in the enrollment process

After the referral is placed, the next set of barriers arises at the CR facility level. We observed that patient outreach was often inconsistent or incomplete. Challenges included outdated contact information, unreturned phone calls, full voicemail boxes, and unsuccessful repeated attempts to connect. Additional delays in insurance approval and limited personnel to manage outreach further complicated the process. Facility capacity constraints also contribute to patient attrition, as described in prior studies reporting increases in space limitations, staff fatigue, and longer waiting periods in the setting of rising referral volumes [19]. These logistical factors collectively contributed to a substantial drop-off between referral and enrollment and highlight the need for more structured and reliable communication processes between the cardiac service line and the CR program.

Predictors of referral

A noteworthy finding was the absence of significant differences in referral rates among physicians, suggesting that system-level influences may outweigh provider-specific behaviors. Our study did not identify strong predictors of either referral or non-referral. Prior work from the NCDR database reported higher referral rates among patients presenting with ST-segment elevation myocardial infarction (STEMI) or non-ST-segment elevation myocardial infarction (NSTEMI) [1]. Although patients with STEMI in our cohort had the highest referral rate (60%, data not shown), the observed lower referral rate among NSTEMI patients contrasts with earlier findings. This discrepancy may be partly attributable to the widespread adoption of high-sensitivity troponin assays in recent years, which has expanded the diagnosis of Type 2 NSTEMI - a population characterized by greater comorbidity and acuity. Several studies have demonstrated that comorbidities are associated with lower CR referral, supporting the possibility that sicker or more clinically complex patients may receive fewer referrals as clinical focus shifts toward acute management rather than long-term secondary prevention [1,8,12,13].

Low enrollment rate

Despite more than half of patients receiving referrals, enrollment into CR remained strikingly low, consistent with reports from other settings. For example, the Swedish registry found that 48% of patients referred after acute myocardial infarction (AMI) enrolled in CR, which is comparable to our finding of 35% enrollment among referred patients [3]. Prior studies have identified logistical obstacles such as distance, transportation, comorbidity burden, and insurance coverage as major barriers to enrollment [20]. Earlier analyses from the NCDR database identified private insurance as a barrier [1], whereas in our study, patients with private insurance had the highest enrollment rates, likely reflecting sample size differences or regional variability. Predictors of CR enrollment vary widely in the literature, underscoring the multifactorial and context-dependent nature of participation. When we examined patient-reported reasons for not enrolling in CR, lack of knowledge about the program emerged as the most common barrier. Prior studies have similarly demonstrated that targeted patient education can significantly increase CR participation; Grace et al. reported a 45% increase in enrollment when a liaison provided structured CR education in addition to placing the order [10]. Patient motivation has also been identified as a critical factor influencing CR engagement [14,21]. Collectively, these findings reinforce the importance of a coordinated and patient-centered approach to CR education and follow-up.

Placing the order at discharge: challenging current metrics

Currently, placing a referral order at discharge is a quality metric monitored by both the NCDR and CMS, and originates from the Get With The Guidelines initiative developed by the American Heart Association in 2009 [22]. Our findings suggest that this metric alone may be insufficient as an indicator of high-quality CR care because referral does not guarantee enrollment or participation. Since improved clinical outcomes are linked to CR participation rather than referral itself, it may be beneficial for quality metrics to evolve toward measuring completed enrollment or meaningful engagement. In this project, referrals placed at discharge tended to result in lower enrollment compared with those placed during the first clinic visit, highlighting that referral timing must be considered within the broader context of patient readiness and health system processes.

Study strengths and limitations

This study has several strengths. It examines the full trajectory from PCI to CR enrollment, combining retrospective EHR review with prospective patient interviews. Reasons for non-participation were documented using predefined categories and standardized questionnaires, enhancing reliability. Data collection was performed within three months of PCI, providing a meaningful window for CR engagement while reducing recall bias. The integration of process-level detail, such as contact attempts, communication failures, and operational barriers, adds practical insight that may inform system-level improvements.

The study also has important limitations. Its single-center design limits generalizability, and the modest sample size may reduce the ability to detect subtle predictors of referral or enrollment. Although 87.5% of non-enrolled patients participated in interviews, the remaining non-responders may differ systematically from responders, introducing potential selection or response bias. Patient-reported reasons for non-participation relied on recall and may be influenced by information or social desirability bias. Because this was an observational quality improvement project, residual confounding is likely, and causal inferences, particularly regarding the relationship between referral timing and enrollment, cannot be assured. Unmeasured factors such as illness severity, intensity of discharge education, transportation access, socioeconomic constraints, and health literacy were not systematically captured but may substantially influence CR participation. Misclassification is also possible due to variation in EHR documentation and differences in how contact attempts were recorded by the CR program. Additionally, local CR program capacity may have affected enrollment and further limited external validity. Given the small sample size, stratified or propensity-adjusted analyses were not feasible but would strengthen the evaluation of referral timing effects in future studies. Finally, given the exploratory nature of this project and the sample size, formal sensitivity analyses were not feasible; future larger studies could incorporate such analyses to better evaluate the observed associations.

Future directions and system changes

Our study demonstrates that, despite more than a decade of reports emphasizing low CR referral and enrollment rates, progress remains limited. A more comprehensive systems-based approach is needed to improve both referral and participation. Strategies such as standardized referral protocols, integrated prompts within the discharge workflow, improved patient and physician education, and reinforcement of CR recommendations during follow-up may collectively enhance engagement. Structured educational materials and consistent messaging may help address the widespread knowledge gaps identified in this study. Strengthening communication between hospitals and CR facilities, and incorporating navigators or coordinators to follow up on referrals, may help close gaps in the referral-to-enrollment pathway. At a broader level, CR programs continue to face space and staffing limitations, underscoring the need for supportive system infrastructure. Future research should explore implementation strategies aimed at improving the full CR pathway and evaluate scalable models such as home-based or virtual CR programs to expand access and support sustained participation.

## Conclusions

This quality improvement project demonstrates that significant gaps remain in both referral and enrollment into cardiac rehabilitation after PCI. Although more than half of eligible patients received a referral, only a small proportion ultimately enrolled, reflecting multiple breakdowns throughout the referral-to-enrollment pathway. The most prominent barriers included lack of patient awareness, inconsistent communication from CR facilities, and logistical challenges related to contact, insurance, and program capacity. These findings suggest that referral alone, particularly when placed at discharge, is insufficient to ensure meaningful participation. These findings should be interpreted as correlational and hypothesis-generating, as unmeasured factors such as illness severity, patient readiness, and variability in discharge education may also contribute to differences in enrollment. Improving CR utilization will require a more coordinated system that emphasizes clear patient education, streamlined workflows, reliable communication between hospital and CR teams, and reinforcement of CR recommendations during early follow-up care. Broader implementation of structured referral protocols, dedicated navigators, and innovative program models such as home-based or virtual CR may help bridge existing gaps and support higher enrollment and engagement. Continued efforts are needed to refine institutional processes and align quality metrics with outcomes that more accurately reflect patient participation and long-term secondary prevention.
